# Fetal volume measurements with three dimensional ultrasound in the first trimester of pregnancy, related to pregnancy outcome, a prospective cohort study

**DOI:** 10.1186/1471-2393-12-38

**Published:** 2012-05-28

**Authors:** Nicol AC Smeets, Marc Prudon, Bjorn Winkens, S Guid Oei

**Affiliations:** 1 Department of Gynecology and Obstetrics, Atrium Medical Centre, Parkstad, Henri Dunantstraat 5, Heerlen, 6401 CX, The Netherlands; 2 Department of General Practice, University of Maastricht, Maastricht, The Netherlands; 3 Department of Methodology and Statistics, University of Maastricht, Maastricht, The Netherlands; 4 Department of Gynecology and Obstetrics, Máxima Medical Centre, Eindhoven-Veldhoven, The Netherlands; 5 Department of Electrical Engineering, Eindhoven University of Technology, Eindhoven, The Netherlands

## Abstract

**Background:**

First trimester growth restriction is associated with an increased risk of adverse birth outcomes (preterm birth, low birth weight and small for gestational age at birth). The differences between normal and abnormal growth in early pregnancy are small if the fetal size is measured by the crown-rump-length. Three-dimensional ultrasound volume measurements might give more information about fetal development than two-dimensional ultrasound measurements. Detection of the fetus with a small fetal volume might result in earlier detection of high risk pregnancies and a better selection of high risk pregnancies.

**Methods:**

A prospective cohort study, performed at the Máxima Medical Centre, in Eindhoven-Veldhoven, the Netherlands. During the routine first trimester scan with nuchal translucency measurement 500 fetal volumes will be obtained. The gestational age is based on the first day of the last menstrual period in a regular menstrual cycle and by the crown-rump-length. The acquired datasets are collected and stored on a hard disk for offline processing and volume calculation. The investigator who performs the volume measurements is blinded for the results of the first trimester scan. The manual mode will be used to outline the Region Of Interest, the fetal head and rump, in all cross sections. The fetal volumes are calculated with a rotational step of 9°.

First, the relation between fetal volume and gestational age, for a set of participants with normal pregnancies (training set), will be assessed. This model will then be used to determine expected values of fetal volume for a normal pregnancy, which will be referred to as expected normal values. Secondly, for a new set of participants with normal pregnancies and a set of participants with complicated pregnancies (together defined as validation set), the observed fetal volumes (FV_observed_) are compared with their expected normal values (FV_expected_) and expressed as a percentage of the expected normal value. The mean difference in percentage error between the set of normal versus complicated pregnancies will then be compared using the independent-samples t-test. Finally, logistic regression analysis will be applied to the validation set of participants to analyze the possibility of predicting the pregnancy outcome after fetal volume calculation in the first trimester, using this percentage error.

**Discussion:**

After this study it is clear whether FV measurement in the first trimester can detect high risk pregnancies. If it is possible to detect these pregnancies, more intensive follow up in these pregnancies might result in fewer complicated pregnancies and fewer fetal morbidities.

## Background

Preterm birth is a growing public health problem that has significant consequences for families. Preterm birth accounts for 12.5% of all births in the United States. The costs for society are at least $26 billion dollar a year [1]. Low birth weight (<2500 g) and birth weight that is small for gestational age (SGA) are associated with increased morbidity and mortality perinatal and in later life [2].

There is a growing body of evidence that complications in pregnancy are the result of the intra-uterine conditions in the first trimester of pregnancy. Monitoring fetal growth during the first trimester of pregnancy is expected to be of significant value in assessing complications in pregnancy. Smith et al. was the first to report about the relationship between first trimester fetal two-dimensional ultrasound (2DUS) measurements in relation to an increased risk of preterm birth, a low birth weight or being small for gestational age (SGA) at birth [3]. Bukowski et al. confirmed the relation between slow growth in the first trimester of pregnancy and a low birth weight [4]. These pregnancies were a result of assisted reproductive technologies excluding delayed ovulation as an explanation for the findings. They also report that a delayed implantation would result in a longer duration of pregnancy. However they found the opposite association, confirming that the delayed implantation could not explain the observed associations [4]. Mook-Kanamori et al. recently confirmed these earlier reports. They reported that fetal growth below the 20^th^ percentile in the first trimester of pregnancy is associated with an increased risk of adverse birth outcomes such as preterm birth, low birth weight and SGA [5].

The differences between normal and abnormal growth in early pregnancy are small if the fetal size is measured by the crown-rump-length (CRL). There are several different definitions used in earlier reports for small fetal size in the first trimester in relation to complicated pregnancies. Abnormal growth is calculated as a difference in expected fetal size (according to the last menstrual period) minus the calculated fetal size by ultrasound (CRL/BPD) in days, ranging from −1 to −8-10 days [3,6,7]. The weakness of all these results is the low positive predictive value, therefore it is not a good screening test in order to select the high risk pregnancies.

Three-dimensional ultrasound (3DUS) volume measurements might give more information about fetal development than 2DUS measurements. Fetal volume (FV) measurements were subject of earlier studies, where the volume measurements proved to be reliable and reproducible [8,9], even in twins [10,11]. A significant correlation between FV and the crown-rump-length (CRL) is already confirmed, with an up to 35-fold increase of the FV and a 4.5-fold increase of the CRL in the first trimester of pregnancy [8-10,12,13]. As the FV rises 7 times faster than the CRL, it can be expected that slight abnormalities in the CRL will be more obvious in FV measurements. Falcon et al. reported that the chromosomal abnormal fetus has a significant smaller FV than the chromosomal normal fetus whereas the CRL in trisomy 21 and Turner syndrome were normal [9,13]. These findings suggest that it is possible to detect early growth impairment with FV measurements, in cases with a normal CRL. Detection of the fetus with a small FV might result in earlier detection of high risk pregnancies

A longitudinal follow up study is necessary in order to obtain this knowledge.

The objective of this study is to determine whether it is possible to detect a fetus at risk for preterm birth and/or low birth weight for gestational age by measuring the fetal volume with three-dimensional ultrasound in the first trimester of pregnancy.

## Methods

This is a prospective cohort study, performed at the Máxima Medical Centre, a teaching hospital in Eindhoven-Veldhoven, the Netherlands.

### Recruitment

500 Participants who have an appointment for the routine first trimester ultrasound scan with nuchal translucency measurement are asked to participate in this study. Inclusion criteria: Singleton pregnancy, age > 18 years, gestational age between 11^+0^ and 13^+6^ weeks. Exclusion criteria: Multiple pregnancy and an uncertain gestational age. An uncertain gestational age is defined as an unknown date of the first day of the last menstrual period and a varying or unknown length of the menstrual cycle. Participants are included after signing an informed consent form. Each participant fills out a questionnaire about their general and obstetric history. The participant fills another questionnaire after the delivery. The participants are referred from other surrounding hospitals and midwives for the first trimester ultrasound scan and the nuchal translucency measurement, therefore this is a low risk population.

### Fetal imaging

The Kretz Voluson 730 3D ultrasound scanner (General Electrics Kretz, Zipf, Austria) is used with the RAB4-8P wide band convex volume probe, a real time 4D-broadband electronic curved-array transducer with a frequency range of 4–8 MHz. The angle sweep is 75^0^. The three-dimensional volumes are acquired during the standard abdominal first trimester scan by an investigator certified for nuchal translucency (NT) measurements. The gestational age is established by menstrual dates and confirmed by routine fetal biometry.

For volume acquisition the fetus has to be motionless during scanning.

### Data acquisition

The routine first trimester (abdominal) ultrasound scan and NT-measurement are performed according to international guidelines [14,15]. 3D View^TM^ (General Electronics, Sonoview II) is used to receive, store digitally and measure the fetal volumes from the 3DUS-datasets. After obtaining the ideal (midsagittal) plane for NT-measurement, an automatic 3DUS sweep is performed, which consists of multiplanar and surface reconstruction modes. The acquired datasets are collected and stored on a hard disk for offline processing and volume calculation. The investigator who performs the volume measurements is blinded for the results of the first trimester scan.

The VOCAL imaging software (an extension of 3D View^TM^) consists of several available modes for volume calculations, the “manual mode” is most frequently used. This mode is more flexible as it employs to manually define the contours of the object of interest with a computer mouse. As the human embryo has an irregular shape, the manual mode will be used to outline the Region Of Interest (ROI), the fetal head and rump, in all cross sections. It is not possible to include the fetal extremities in these measurements, because the software does not allow to define separate structures in one cross section. Therefore the ROI has to consist of one continuous object in every cross section. This method was also used by Falcon et al. [9,13].

With VOCAL it is possible to use four different rotational steps that define the angle through which the object of interest is rotated. These steps are 6°, 9°, 15° or 30°, which results in respectively 30, 20, 12 or 6 cross sections for each volume measurement, as the dataset is rotated 180^0^ to complete one volume measurement.

The fetal volumes are calculated with a rotational step of 9**°** in the A(axial)-plane, which is a longitudinal plane. This 9**°** rotation is to be preferred in irregular objects, as it is as reliable as the 6**°** rotation, but significantly faster to perform [16].

**Figure 1:**
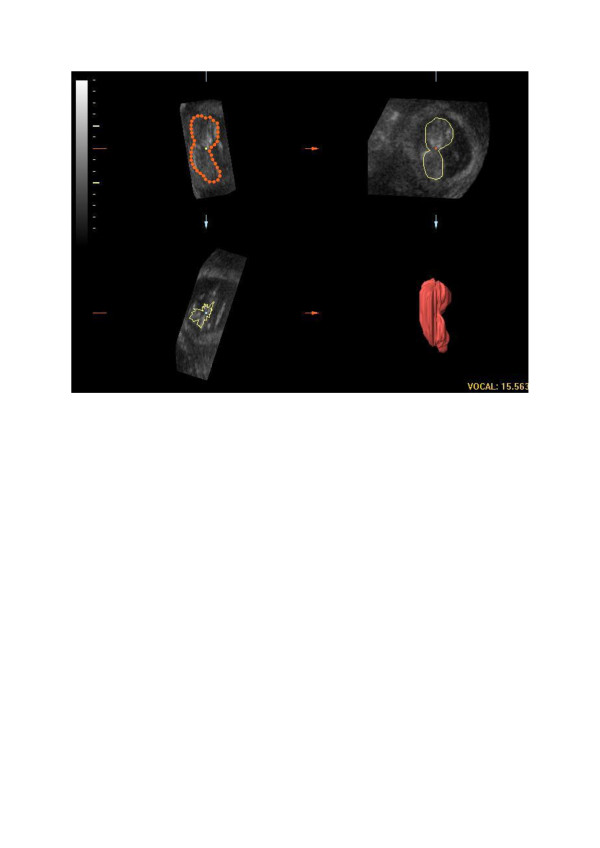
An image of a fetal volume reconstruction.

The 9**°** rotational step results in a sequence of 20 longitudinal sections of the fetus around a fixed axis. In each of these planes the two-dimensional (2D) contour of the fetus (excluding the limbs) is defined manually, as described by others [10,11,13]. The VOCAL program then calculates the volume of the defined contour. After calculation the computed reconstruction of the fetus is displayed together with the fetal volume (Figure 1). There can be an undulating surface of the 3D image, which is caused by the rotational steps and represents the ROI’s in each measured plane. We have previously reported that the inter- and intraobserver agreement of fetal volume measurements by 3DUS is very high [17].

### Statistical analysis

First, for a set of participants with normal pregnancies (training set), the relation between fetal volume and gestational age (GA) will be assessed using multiple linear regression. The influence of fetal gender and parity will be observed, if needed different training sets will be generated. This model will then be used to determine expected values of fetal volume for a normal pregnancy, which will be referred to as expected normal values. Secondly, for a new set of participants with normal pregnancies and a set of participants with complicated pregnancies (together defined as validation set), the observed fetal volumes (FV_observed_) are compared with their expected normal values (FV_expected_) and expressed as a percentage of the expected normal value, i.e. percentage error = 100% * (FV_observed_ – FV_expected_)/FV_expected_. The mean difference in percentage error between the set of normal versus complicated pregnancies will then be compared using the independent-samples t-test. Finally, logistic regression analysis will be applied to the validation set of participants to analyze the possibility of predicting the pregnancy outcome after fetal volume calculation in the first trimester, using this percentage error. For the statistical analysis SPSS^TM^ (Chicago Illinois, USA) version 18.0 for Windows will be used. A two-sided p-value ≤ 0.05 is considered statistically significant. As we use a smaller rotational angle, there might be a difference with the results of Falcon et al. [9].

### Sample size

At the routine first trimester scan with nuchal translucency measurement, 500 fetal volumes will be measured. 50% of these volumes (n = 250) will be used as the training set and the other 50% as the validation set. Since it was expected that at least 10% of all pregnancies will be complicated by pre-term delivery or a low birth weight for gestational age, the validation set consists of 50 complicated pregnancies and 200 normal pregnancies. Based on the independent-samples t-test to compare the mean percentage error between complicated and normal pregnancies, these sample sizes are sufficient to detect a medium standardized effect size (Cohen’s d = 0.5) with sufficient power (80%) using a significance level of 5% and accounting for 10-20% lost to follow up.

The protocol is approved by the hospital’s medical ethics committee and informed consent will be obtained prior to inclusion in this study.

## Discussion

After this study it is clear whether FV measurement in the first trimester can detect high risk pregnancies. If it is possible to detect these pregnancies, more intensive follow up in these pregnancies might result in less complicated pregnancies and less fetal morbidity.

We expect to perform measurements in a normal population. Based on the definition of SGA, it is to be expected that 50 fetuses will be SGA (10%), and 62 deliveries will be preterm (12,5%).

## Summary

This study is designed to provide information on whether FV volume measurements with three-dimensional ultrasound in the first trimester of pregnancy can be helpful in selecting high risk pregnancies.

## Competing interest

The authors declare that they have no competing interests.

## Authors’ contributions

NS and SO designed the study with input from MP and BW. NS, MP, BW and SO drafted the manuscript. NS, MP, BW and SO produced the detailed working protocol. All authors read and approved the final manuscript.

## Pre-publication history

The pre-publication history for this paper can be accessed here:

http://www.biomedcentral.com/1471-2393/12/38/prepub
